# Impacts of N-P-K-Mg Fertilizer Combinations on Tree Parameters and Fungal Disease Incidences in Apple Cultivars with Varying Disease Susceptibility

**DOI:** 10.3390/plants13091217

**Published:** 2024-04-28

**Authors:** Ádám Csihon, István Gonda, Marianna Sipos, Imre J. Holb

**Affiliations:** 1 Institute of Horticulture, Faculty of Agricultural and Food Sciences and Environmental Management, University of Debrecen, Böszörményi Str. 138, 4032 Debrecen, Hungary; csihonadam@agr.unideb.hu (Á.C.); gonda@agr.unideb.hu (I.G.); siposmarianna@agr.unideb.hu (M.S.); 2 Eötvös Loránd Research Network (ELKH), Centre for Agricultural Research, Plant Protection Institute, Herman Ottó út 15, 1022 Budapest, Hungary

**Keywords:** N-P-K-Mg nutrient, fertilization, apple, apple scab, powdery mildew, yield parameters, correlation, linear regression

## Abstract

Adequate mineral fertilization helps to ensure optimal tree growth, fruit development, and predictable yield of apple trees. This 7-year study (2016–2022) aims to investigate the effect of nitrogen (N), phosphorus (P), potassium (K), and magnesium (Mg) fertilizer combinations (NP, NPK, NPKMg, and control) on eight parameters (trunk cross-sectional area—TCSA; fruit yield—FY; number of fruit per tree—FNT; crop load—CL; fruit diameter—FD; fruit weight—FW; fruit scab incidence—FSI; and powdery mildew incidence on shoot—PMIS) on the cultivars (cvs) ‘Golden Reinders’ (disease susceptible) and ‘Pinova’ (scab and mildew tolerant). In the 7-year period, TCSA values continuously increased for both cultivars over the years. Fertilizer treatments showed significant differences on TCSA but the effect varied greatly annually among fertilizer treatments. Fertilizer treatments had increasing effects on FY and FNT in 2018 and 2022, on CL in 2018, on FD in 2018 and 2019, and on FW in 2016 and 2018 in both cultivars compared to the control treatment. FSI values were the lowest in the NPKMg treatment for cv. ‘Golden Reinders’ in 2016, 2017, and 2022; for cv. ‘Pinova’ in 2016; PMIS values for cv. ‘Golden Reinders’ in 2017, 2018, 2021, and 2022; and for cv. ‘Pinova’ in 2018. Correlation and regression analyses revealed strong and significant (*p* = 0.05) relationships between FNT versus (vs.) TCSA, FNT vs. FY, FW vs. TCSA, CL vs. FY, FW vs. FD, and FSI vs. FW. In conclusion, our study showed that multiyear application of fertilizer combinations can successfully increase TCSA and yield parameters as well as reduce fungal disease incidences, especially on the disease-susceptible cultivar in sandy soil with moderate fertility, under Central-European continental climate conditions.

## 1. Introduction

Apples are one of the largest fruit crops in temperate climates, with worldwide production showing a growing trend that exceeded 90 million tons in 2022 [1]. A key component of apple production technology is the provision of adequate nutrients to the trees through mineral fertilization, which ensures optimal growth, development, and predictable productivity of apple trees [2,3,4].

Mineral fertilizers provide essential nutrients in the soil and ensure an adequate nutrient supply, including nitrogen (N), phosphorus (P), potassium (K), calcium (Ca), magnesium (Mg), and others. These nutrients play crucial roles in both the vegetative and generative development of apple trees [2,5,6,7,8,9,10,11,12]. Proper nutrient supply via mineral fertilization promotes balanced nutrient uptake by the tree and efficient photosynthesis in plant organs [13,14,15].

Vegetative growth is greatly enhanced by nitrogen fertilization [10,16,17,18,19,20]. Nitrogen is a fundamental component of amino acids, proteins, and chlorophyll, all of which are crucial for cell division, elongation, and photosynthesis. Adequate nitrogen supply stimulates the production of new shoots and leaves, thereby increasing tree size and canopy development [10,16,17,18,19,20]. Phosphorus, on the other hand, plays a vital role in root growth and branching development [21,22,23]. Well-developed roots enhance nutrient and water uptake, providing a solid foundation for tree growth and stability [10,18].

Flowering and fruit set in apple trees are highly dependent on phosphorus and potassium, which are crucial for the initiation and development of flower buds. These nutrients play significant roles in the processes of flowering, pollination, and subsequent fruit set [19,20,21,22,23]. Potassium, in particular, is essential for fruit size and quality [24,25]. The demand for potassium in apple trees peaks during ripening [24,25]. Deficiencies in phosphorus and potassium can result in reduced flower formation, poor pollination, and low fruit set, ultimately leading to a decrease in overall fruit quantity [14,15,19,20,21,22,23,24,25].

Adequate nutrient levels ensure optimal fruit development (size and weight) and reduce premature fruit drop [14,18,26,27,28] as well as support the accumulation of carbohydrates and other compounds in the fruit [15,17,21].

Optimal uptake of NPK and Mg nutrients by apple trees increases tolerance to diseases and pests, while imbalances or deficiencies in nutrients can lead to reduced photosynthesis and increased susceptibility to diseases and pests [26,29,30]. Certain nutrients, such as potassium and calcium, help strengthen cell walls and improve the tree’s resistance to diseases. However, N excess often increases the susceptibility of plants to fungal diseases [31,32,33,34,35,36,37,38]. Increasing N fertilization was shown to enhance powdery mildew development on strawberries [36] and dogwoods [34]. In the case of apples, an excess of soil-applied nitrogen was demonstrated to increase disease development caused by *Neonectria ditissima* [37,38]. Nitrogen excess induces vigorous shoot growth in apple trees, which elevates the susceptibility of shoots to *Podosphaera leucotricha* [35,38] and reduces leaf resistance to *Venturia inaequalis* [31,32].

Although numerous studies have evaluated nutrient supply for apple vegetative and generative parameters, the effect of nutrient supply on apple tree vegetative and generative parameters, along with fungal diseases in tolerant/resistant and susceptible cultivars, has rarely been investigated in long-term multiyear studies. Additionally, correlations among tree parameters and disease incidences have not been explored, but such analysis may help us better understand the influence of nutrient supply on fungal diseases in relation to tree parameters.

This 7-year study aims to investigate the effect of four fertilizer treatments including nitrogen, phosphorus, potassium, and magnesium fertilizer combinations (NP, NPK, NPKMg, and control) on six tree parameters (trunk cross-sectional area, fruit yield, fruit number, crop load, fruit size, fruit weight) and on the incidences of apple scab on fruit and powdery mildew on shoots. The experiment was conducted on the disease-susceptible cultivar (cv.) ‘Golden Reinders’ and on the scab and powdery mildew-tolerant cv. ‘Pinova’.

## 2. Materials and Methods

### 2.1. Study Area, Plant Material, Orchard Management, and Meteorological Assessment

A seven-year study from 2016 to 2022 was performed in an experimental apple orchard of the University of Debrecen, Pallag Experimental Station, Debrecen–Pallag, Hungary (47°35′31.5″ N, 21°38′19.3″ E). Soil properties of the experimental site are summarized in Table 1. The soil type is light and sandy with low humus content. The soil is slightly alkaline (pH 7.5–7.6). The nitrogen of the soil was lower than the optimum, while phosphorus, potassium, and magnesium in the topsoil were optimal for the standard sandy soil type (Table 1), according to the Agricultural Technical Guidelines [39].

The experimental orchard was established in the spring of 2006. Trees were grafted on M.26 rootstock and trained to a slender spindle canopy (4.0 × 1.5 m = 1667 trees ha^−1^). Before the experiment, annual NPK fertilization was applied at rates of 30–40–50 kg ha^−1^ from 2006 onwards. At the commencement of the first year of experimentation in 2016, the trees were 10 years old with an average height of 3.5 m. Two apple cultivars were evaluated: the disease-susceptible cultivar ‘Golden Reinders’ [40], and the apple scab and powdery mildew-tolerant cultivar ‘Pinova’ [41].

**Table 1:** Seven soil parameters in the experimental site (Debrecen–Pallag, Hungary, 2016). Optimal values are given for sandy soil according to the Agricultural Technical Guidelines [39].

Soil Parameters	0–20 cm	20–40 cm	40–60 cm	Optimal Value
**Humus content (%)**	0.9	0.9	0.8	1.2–2.0
**pH**	7.6	7.6	7.5	5.7–7.6
**NO_3_ + NO_2_^−^ − N (mg kg^−1^)**	4.01	3.65	2.86	8.0–10.0
**AL-P_2_O_5_ (mg kg^−1^)**	540	465	373	80
**AL-K_2_O (mg kg^−1^)**	340	464	308	100–120
**AL-Mg (mg kg^−1^)**	177	189	184	60
**CaCO_3_ (m/m) %**	0.33	0.35	0.21	<3%

In the soil samples, the AL-soluble content of P, K, Ca, and Mg was determined according to Egnér et al. [42]. The measurement of NO_3_^−^ + NO_2_^−^ − N content was performed according to Skalar [43]. CaCO_3_, content was determined by the methods of Filep [44], using a Scheibler-type calcimeter.

Orchard management was carried out according to the European Integrated Fruit Production (IFP) guidelines [45]. The plantation was equipped with a drip irrigation system. Winter pruning was performed once a year, in February. Mechanical fruit thinning was conducted annually in mid-June for cv. ‘Pinova’, whereas it was only carried out in 2016, 2018, 2020, and 2022 for cv. ‘Golden Reinders’, owing to reduced fruit set caused by spring frosts in 2017 and 2021. Concurrently, chemical thinning was applied in mid-May using 6-benzyladenine (Globaryll-100, Globachem Nv, Sint-Truiden, Belgium) during these years.

Meteorological parameters (mean temperature, minimum temperature, and precipitation) were recorded daily from January 2016 to December 2022 by the Metos Agrometeorological Station located in the experimental station.

### 2.2. Fertilizer Treatments

Four mineral fertilization treatments (NP, NKP, NKPMg, and control) with four active ingredients (nitrogen—N; phosphorus—P_2_O_5_; potassium—K_2_O; and magnesium—MgO) were applied in the experimental apple orchard for 7 years from 2016 to 2022 (Table 2). The two cultivars ‘Pinova’ and ‘Golden Reinders’ belong to the same category in terms of growth vigor, with the achievable yield being 60–70 t ha^−1^. The nutrient requirements of the two cultivars can be considered identical [46]. The dosages of the four active ingredients were applied with the following application rates: N (60 kg ha^−1^), P (80 kg ha^−1^), K (100 kg ha^−1^), and MgO (30 kg ha^−1^). Control trees did not receive fertilizers during the 7-year evaluation period. For nitrogen supply, Genezis Pétisó mineral fertilizer (Genezis Ltd., Pétfürdő, Hungary) containing 27% nitrogen active ingredients was applied. For phosphorus supply, Genezis szuperfoszfát mineral fertilizer (Genezis Ltd., Pétfürdő, Hungary) was applied, which had 18% active P_2_O_5_. Potassium was supplied by Genezis Kálisó (Genezis Ltd., Pétfürdő, Hungary) mineral fertilizer, containing 60% active K_2_O, while magnesium supply was provided by Keserűsó Espo Top mineral fertilizer, containing 16% active MgO.

For each fertilizer treatment and cultivar, seven trees were selected as assessment plots and replicated four times. Subsequently, the middle five trees were chosen for assessments within each plot. The first and seventh trees in the plots served as buffer trees without assessment. The application timing of the fertilizers is summarized in Table 3. Nitrogen fertilizer was always applied in spring due to the risk of nutrient leaching during winter. Fertilizers were applied as soil application at a distance of 1 m from the tree trunk on both sides of the trees on an annual basis. Fertilizers were incorporated into the soil at a shallow depth. The soil in the control treatment was also disturbed to the same shallow depth, without fertilizer application.

### 2.3. Assessment of Six Tree Parameters

Six tree parameters were assessed: trunk cross-sectional area (TCSA), fruit yield (FY), number of fruit per tree (FNT), crop load (CL), fruit diameter (FD), and fruit weight (FW). For each cultivar, 5-tree replicates per assessment plot were selected for assessments of all parameters.

Trunk thickness was measured after leaf fall in November each year with a Vernier caliper at the trunk halfway between the graft union and the main scaffold branches. From trunk thickness, TSCA was calculated in cm^2^. For TSCA, the difference in each year was also calculated, with 2016 as the reference point (0), and then Y2-Y1, Y3-Y2, Y4-Y3, Y5-Y4, Y6-Y5, and Y7-Y6.

Fruit picking was performed at the biological maturity stage of both cultivars, determined by the starch–iodine test. Harvest dates were 18, 15, 13, 4, 23, and 21 September in 2016, 2017, 2018, 2019, 2020, and 2022, respectively, for cv. ‘Golden Reinders’, as well as 3 October 2016, 27 September 2017, 29 September 2018, 1 October 2019, 7 October 2020, 29 September 2021, and 28 September 2022 for cv. ‘Pinova’. Fruit yield (kg tree^−1^) and number of fruit per tree were measured by weighing/counting all the fruit on each selected tree. Crop load was calculated as fruit yield divided by trunk cross-sectional area (kg cm^−2^). Fruit diameter (mm) was assessed with a Vernier caliper based on 20 fruits per tree (100 fruits per treatment). Fruit weight (g) was measured using the same 100 fruit with a digital scale, with a precision of two decimal places.

### 2.4. Assessment of Incidences of Apple Scab and Apple Powdery Mildew

**Figure 1:**
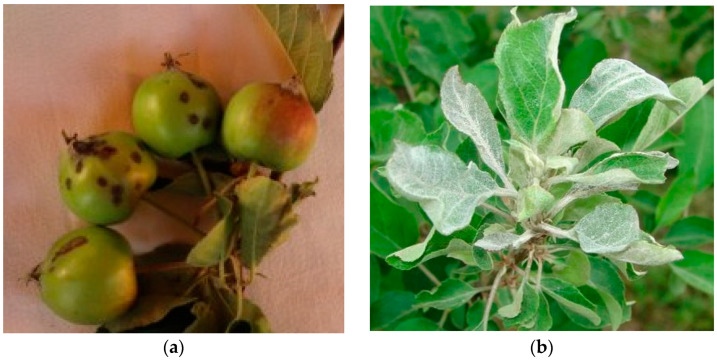
Representative symptoms of apple scab on fruit (**a**) and representative symptoms of powdery mildew on shoot (**b**). Photos by I.J. Holb.

Apple scab [*Venturia inaequalis* (Cooke) G. Winter] assessments were conducted on fruit for each fertilizer treatment, cultivar, and year. For fruit assessment, 25 fruits with typical cultivar characteristics were observed on each quadrant of a selected tree at harvest in each year (4 × 25 fruits per tree and 5 × 4 × 25 fruits per assessment plot). A fruit was considered diseased if at least one visible scab lesion was observed on the fruit (Figure 1a). Fruit scab incidences (FSI) from the four quadrants per tree were averaged to obtain the percentage of diseased fruits per tree. For each cultivar, the same trees were selected for fruit scab assessment as for the other tree parameters, in the years 2016, 2017, 2018, 2019, 2021, and 2022.

Apple powdery mildew [*Podosphaera leucotricha* (Ellis & Everh.) E. S. Salmon] assessments were conducted at harvest on shoots of the same trees used for apple scab and other tree assessments in the same years. All shoots from each quadrant of a tree were examined for disease symptoms. Shoots were considered diseased if at least one leaf was covered with mycelium and/or spores (Figure 1b). Powdery mildew incidences on shoot (PMISs) from the four quadrants per tree were averaged to obtain the percentage of diseased shoots per tree.

### 2.5. Data Analyses

#### 2.5.1. Analysis of Variance (ANOVA)

Data for each parameter were averaged to obtain a single value per tree for each cultivar, fertilizer treatment, and year. The data were subjected to analyses of variance (ANOVA) in order to determine the effect of cultivars, fertilizer treatments, and years, and their interactions were also assessed on the eight parameters. Subsequently, significant F-tests (*p* < 0.05) were followed by a least significant difference (LSD) test for each year, fertilizer treatment, and cultivar in order to compare the means of all parameters using LSD_0.05_ values. Genstat Release 9.1 (Lawes Agricultural Trust, IACR, Rothamsted, UK) was used for the analyses.

#### 2.5.2. Correlation and Linear Regression Analyses

Relationships among all parameters were analyzed by Pearson correlation coefficients (*r*), and their associated significance levels (*p* = 0.05) were separately determined for the two cultivars. Subsequently, the strongest significantly correlated pairs were identified for the two cultivars and the overall data set. Additionally, the strongest significantly correlated pairs were plotted against each other, and linear regression functions were fitted for the four fertilizer treatments. A *t*-test was then used to determine whether the regression slopes were significantly (*p* = 0.05) different among the four fertilizer treatments. For the analyses, Genstat Release 9.1 (Lawes Agricultural Trust, IACR, Rothamsted, UK) was utilized.

## 3. Results

### 3.1. Analysis of Variance

Analysis of variance on the parameters of trunk cross-sectional area, fruit yield, number of fruit per tree, crop load, fruit diameter, fruit weight, and incidences of apple scab and powdery mildew showed significant (*p* < 0.05) effects for fertilizer treatments, cultivars, and years (Table 4). The only exception was the parameters of trunk cross-sectional area, where the effects for cultivars were nonsignificant at the *p* < 0.05 level. Interactions for the treatment effects were nonsignificant for all treatments (Table 4).

### 3.2. Weather Conditions

The monthly mean temperature ranged from −6.0 to 25 °C during the assessed period from January 2016 to December 2022 (Table S1). The lowest minimum temperature values indicated that late spring frost in April (below −2 °C) occurred in 2020, 2021, and 2022. In 2021, cv. ‘Golden Reinders’ suffered total frost damage; accordingly, it was not possible to evaluate all parameters in that year. The annual rainfall ranged from 397 to 720 mm between 2016 and 2022 (Table S1).

### 3.3. Trunk Cross-Sectional Area

In the 7-year period, trunk cross-sectional area (TCSA) values continuously increased for both cultivars with years (Table 5). For cv. ‘Golden Reinders’, TCSA was between 31.4 and 35.6 cm^2^ in 2016, while after seven years, values ranged from 72.9 to 92.9 cm^2^ in 2022. In cv. ‘Pinova’, TCSA was between 32.0 and 48.0 cm^2^ in 2016, while the values ranged from 72.0 to 102.5 cm^2^ in 2022. Although ‘overall (treatments)’ TCSA values were higher for cv. ‘Pinova’ compared to cv. ‘Golden Reinders’, the TCSA values of the two cultivars did not differ significantly at *p* = 0.05.

Significant differences among the four fertilizer treatments were found for cv. ‘Golden Reinders’ in 2019, 2021, and 2022; and for cv. ‘Pinova’ in 2016, 2017, 2018, 2020, 2021, 2022, and ‘overall (year)’ (Table 5). The values of the NPKMg treatment were significantly higher compared to the values of the NP treatment for cv. ‘Golden Reinders’ in 2019, 2020, and 2022, while the two treatments were not significantly different from the other two treatments (control and NPK). In the case of cv. ‘Pinova’, the values of the NP treatment were significantly higher compared to the values of the NPK treatment in all years (with the exception of 2019), while the two treatments were not significantly different from the other two treatments (control and NPKMg).

The overall values for the four fertilizer treatments, ‘overall (cultivars)’, showed that the overall values of the NPKMg treatment were significantly higher compared to the values of the NPK treatment in 2020, 2021, and 2022, while the two treatments were not significantly different from the other two treatments (control and NP) (Table 5).

### 3.4. Fruit Yield and Number of Fruit per Tree

The cultivar ‘Golden Reinders’ suffered from 100% frost damage of fruit in 2021, and partial fruit loss in 2017 and 2019, while cv. ‘Pinova’ provided a more homogenous yield over the assessed period (Table 6). The highest fruit yield was recorded in the NPK treatment in 2022 on cv. ‘Golden Reinders’ (69.1 kg tree^−1^ corresponding to 115.3 t ha^−1^). The ‘overall (treatments)’ fruit yield data showed significant variability in the case of cv. ‘Golden Reinders’ (0–61.3 kg tree^−1^) compared to cv. ‘Pinova’ (29.1–53.1 kg tree^−1^); the fruit yield of the two cultivars was significantly different in 2017 and 2019 at *p* = 0.05 (Table 6).

Significant differences in fruit yield values among the four fertilizer treatments were found for cv. ‘Golden Reinders’ in 2017, 2018, and 2022, and for cv. ‘Pinova’ in 2016, 2018, 2019, and 2022 (Table 6).

The overall values of fruit yield for the four fertilizer treatments, ‘overall (cultivars)’, showed that the overall values of NPK and NP treatment were significantly higher compared to values of the control treatment in 2018 and 2022, respectively, while the two treatments were not significantly different from the other two treatments (NP and NPKMg, and NKP and NKPMg, respectively) (Table 6).

The cultivar ‘Golden Reinders’ had a wide range of number of fruit per tree (between 14 and 612), while cv. ‘Pinova’ provided a more homogenous number of fruit per tree (between 143 and 387) over the assessed period (Table 7). The highest number of fruit per tree was recorded in the NPK treatment in 2020 on cv. ‘Golden Reinders’ (612), with the lowest one in the NPK treatment in 2019 on cv. ‘Golden Reinders’ (14). The ‘overall (treatments)’ data showed that the number of fruit per tree of the two cultivars was significantly different in 2017, 2019, 2020, and 2022 at *p* = 0.05 (Table 7).

Significant differences in the number of fruit per tree were found among the four fertilizer treatments for cv. ‘Golden Reinders’ in all years, and for cv. ‘Pinova’ in 2016, 2018, 2019, and 2022 (Table 7). For example, the values of the NP treatment were significantly different from the NPK treatment for cv. ‘Golden Reinders’ in 2017, 2018, 2019, and 2020. In 2019, the values of the NP and NKPMg treatments for cv. ‘Golden Reinders’ were significantly higher compared to the NKP treatment. In 2022, the values of the NPKMg treatment were significantly higher than the control.

In the case of cv. ‘Pinova’, the values of NP, NKP, and NKPMg treatments were significantly higher compared to the values of the control treatment in 2016; the values of the NP treatment were significantly higher compared to the values of the control treatment in 2018 and 2022; and the values of the NPK treatment were significantly higher compared to the values of the control treatment in 2019 (Table 7).

The overall values of the number of fruit per tree for the four fertilizer treatments, ‘overall (cultivars)’, showed that the overall values of the NPK treatment were significantly higher compared to values of the control treatment in 2016 and 2018, while the two treatments were not significantly different from the other two treatments (NP and NPKMg) (Table 7). In 2022, the overall values of the NPKMg treatment were significantly higher compared to the values of the control treatment, while the two treatments were not significantly different from the other two treatments (NP and NPK) (Table 7).

### 3.5. Crop Load

Similarly to other assessed parameters, cv. ‘Golden Reinders’ had a wide range of crop load values (between 0.05 and 1.25 kg cm^−2^), while cv. ‘Pinova’ provided a more homogenous crop load (between 0.42 and 1.00 kg cm^−2^) over the 7-year period (Table 8). The highest crop load was recorded in 2016 in the NPK treatment for both cultivars: 1.25 kg cm^−2^ for cv. ‘Golden Reinders’ and 1.00 kg cm^−2^ for cv. ‘Pinova’. The lowest values were also found in the NPK treatment in 2019 on cv. ‘Golden Reinders’ (0.05 kg cm^−2^). The ‘overall (treatments)’ data showed that the crop load of the two cultivars was significantly different only in two years (2019 and 2020) at *p* = 0.05 (Table 8).

Values of crop load among the four fertilizer treatments were significantly different for cv. ‘Golden Reinders’ in 2018 and 2019, and for cv. ‘Pinova’ in 2016, 2018, 2019, and ‘overall (year)’ (Table 8). The values of the NPK treatment were significantly higher than the NP and control treatments for cv. ‘Golden Reinders’ in 2018. However, in 2019, the values of the NPK treatment for cv. ‘Golden Reinders’ were significantly lower than the values of the NP the treatment, while the two treatments were not significantly different from the other two treatments (control and NPKMg).

In the case of cv. ‘Pinova’, the values of NKP treatment were significantly higher compared to values of the control treatment in 2016, 2018, 2019, and ‘overall (year)’, while the two treatments were not significantly different from the other two treatments (NP and NPKMg) (Table 8).

The overall values of crop load for the four fertilizer treatments ‘overall (cultivars)’ showed significant differences only in 2018. In this year, the values of the NPK treatment were significantly higher compared to the values of the control treatment, while the two treatments were not significantly different from the other two treatments (NP and NPKMg) (Table 8).

### 3.6. Fruit Diameter and Fruit Weight

During the 7-year period, the mean fruit diameter of both cultivars reached the market requirements of 70 mm, with the exception of cv. ‘Pinova’ in 2021 in the control and NPK treatments (Table 9). The largest fruit (83.0 mm) was harvested in 2016 in the NPKMg treatment for cv. ‘Golden Reinders’, while the smallest one (69.1 mm) in 2021, in the control treatment for cv. ‘Pinova’ (Table 9).

Significant differences in the fruit diameter were found among the four fertilizer treatments for cv. ‘Golden Reinders’ in 2016–2019, and for cv. ‘Pinova’ in 2018, 2019, 2021, and 2022 (Table 9). In the case of cv. ‘Golden Reinders’, the values of the NPKMg treatment were significantly higher than those of the NP treatment in 2016. In 2017, the values of the NPK treatment were significantly higher compared to the NP treatment. In 2018, the values of the NP and NKPMg treatments were significantly higher compared to the control treatment. In 2019, the values of the NPKMg treatment were significantly higher than the other three treatments (Table 9).

In the case of cv. ‘Pinova’, fruit diameter values of the NKPMg treatment were significantly higher compared to the values of the control treatment in 2018; the values of NP and NPKMg treatments were significantly higher compared to the values of the control treatment in 2019; the values of the NP treatment were significantly higher compared to the values of the control treatment in 2020; and the values of NPK and NPKMg treatments significantly differed from the control treatment (Table 9).

The overall values for the four fertilizer treatments ‘overall (cultivars)’ showed that the overall fruit diameter values of NPKMg treatment were significantly higher compared to the values of the control treatment in 2016, 2018, and 2019, while the two treatments were not significantly different from the other two treatments (NP and NPK) (Table 9). In 2021, the overall values of the NP treatment were significantly higher compared to the values of the control treatment, while the two treatments were not significantly different from the other two treatments (NPK and NPKMg).

Similarly to fruit diameter values, the fruit weight was the lowest in 2021 in the control treatment (133 g) for cv. ‘Pinova’, while the highest incidence was observed in 2016 in the NPKMg treatment (238 g) for cv. ‘Golden Reinders’ (Table 10). The ‘overall treatment’ data showed that the fruit weight of the two cultivars was significantly different in 2016, 2017, 2018, and 2020 at *p* = 0.05 (Table 10).

Significant differences in fruit weight were found among the four fertilizer treatments for cv. ‘Golden Reinders’ in 2016–2019, and for cv. ‘Pinova’ in 2016–2022 (Table 10). The values of the NPKMg treatment were significantly higher compared to the other three treatments in 2016. In 2017, the values of the NP treatment were significantly lower compared to the other three treatments. In 2018, the values of the NP and NKPMg treatments were significantly higher compared to the control and NPK treatments. In 2019, the values of the NPK and control treatments were significantly different from the NP and NPKMg treatments (Table 10).

In the case of cv. ‘Pinova’, fruit weight values of the NKPMg treatments were significantly higher compared to values of the control treatment in 2016, 2018, 2019, 2020, and 2021. In 2017, the values of the NPKMg treatment were significantly higher compared to the values of the NP treatment. In 2022, the values of the NPK and NKPMg treatments were significantly different from the control treatment (Table 10).

The overall values for the four fertilizer treatments ‘overall (cultivars)’ showed that the overall values of NPKMg treatment were significantly higher compared to values of the control treatment in 2016, 2018, and 2021 (Table 10). In 2017, the overall values of the NP treatment were significantly lower compared to the values of the NPK and NPKMg treatments. In 2019, the overall values of the NPK treatment were significantly lower compared to the values of the NP and NPKMg treatments.

### 3.7. Incidences of Apple Scab and Apple Powdery Mildew

Apple scab incidence on fruit was the smallest in 2018, 2019, and 2021 on cv. ‘Pinova’ in all four fertilizer treatments (0%), while the largest one was observed in 2016 on cv. ‘Golden Reinders’ in the NP treatment (14.0%) (Table 11). The ‘overall (treatments)’ data showed that the fruit scab incidence of cv. ‘Golden Reinders’ was significantly higher compared to the values of cv. ‘Pinova’ in 2016–2019 and 2022 at *p* = 0.05 (Table 11).

Significant differences in fruit scab incidence were found among the four fertilizer treatments for cv. ‘Golden Reinders’ in 2016, 2017, and 2022, and for cv. ‘Pinova’ in 2016 (Table 11). The values of the NPKMg treatment were significantly lower compared to the control treatment in 2016 for both cultivars. In 2017, the values of the NPKMg treatment were significantly lower compared to the control and NP treatments for cv. ‘Golden Reinders’. In 2022, the values of the NPKMg treatment were significantly lower compared to the control treatment for cv. ‘Golden Reinders’ (Table 11).

The overall values of fruit scab incidence ‘overall (cultivars)’ showed that the value of the NPKMg treatment was significantly lower compared to the values of the control and NP treatments in 2016 and to the value of the control treatment in 2017 (Table 11).

Powdery mildew incidence on shoots was the lowest (1.3%) in 2016 on cv. ‘Pinova’ in the NKPMg treatment, while the highest incidence (7.3%) was observed in 2018 on cv. ‘Golden Reinders’ in the control treatment (Table 12). The ‘overall (treatments)’ data showed that the powdery mildew incidence of cv. Golden Reinders’ was significantly higher compared to the values of cv. ‘Pinova’ in 2016–2019, 2021, and 2022 at *p* = 0.05 (Table 12).

Significant differences in powdery mildew incidence were found among the four fertilizer treatments for cv. ‘Golden Reinders’ in 2017, 2018, 2021, and 2022, and for cv. ‘Pinova’ in 2018 (Table 12). The disease incidence of the NPKMg treatment was significantly lower compared to the control treatment in 2017, 2018, 2021, and 2022 for cv. ‘Golden Reinders’ and for cv. ‘Pinova’ in 2018 (Table 12).

The values of powdery mildew incidence in ‘overall (cultivars)’ for the four fertilizer treatments showed that the overall value of the NPKMg treatment was significantly lower compared to the value of the control treatment in 2018 (Table 12).

### 3.8. Correlation among Parameters and Linear Regression

Pearson correlation coefficient (*r*) was the highest (*r* = 0.95) between FNT and FY in the overall data analyses including both cultivars (Table 13). Six correlation pairs were significant (*p* = 0.05) in the overall data set and for each cultivar; five parameter pairs correlated positively (FNT vs. TCSA, FNT vs. FY, CL vs. FY, FW vs. FD, and FSI vs. FW), and one negatively (FW vs. TCSA) (Table 13). In the case of cv. ‘Pinova’, three additional correlation pairs (FY vs. TSCA, FD vs. FNT, and FW vs. FNT) also showed strong and significant (*p* = 0.05) relationships (Table 13). In the case of cv. ‘Golden Reinders’, two additional correlation pairs (CL vs. FNT and FD vs. TCSA) also showed strong and significant (*p* = 0.05) relationships (Table 13).

**Figure 2:**
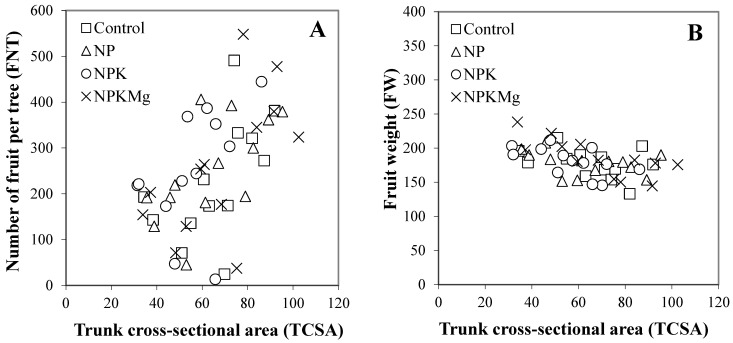

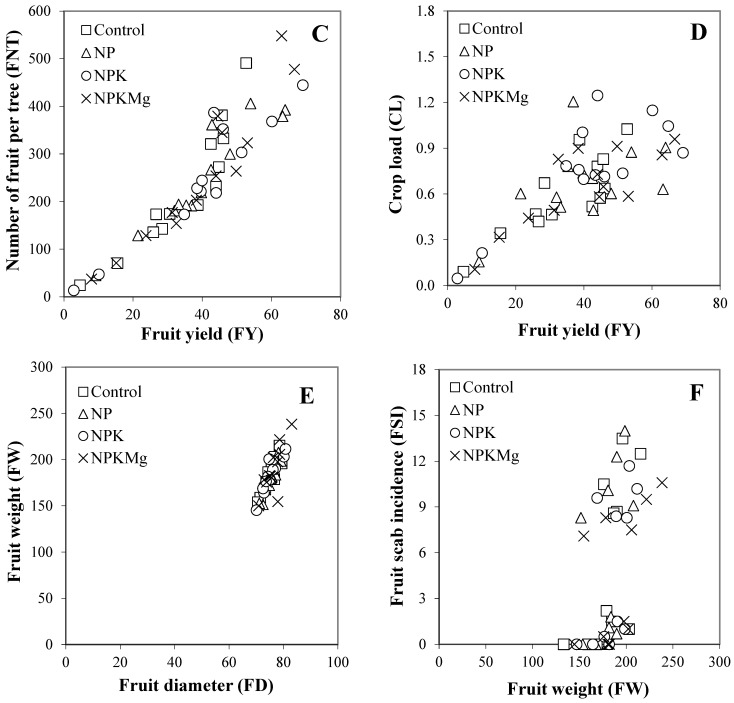
Relationships between 6 variable pairs: FNT versus (vs.) TCSA (**A**), FW vs. TCSA (**B**), FNT vs. FY (**C**), CL vs. FY (**D**), FW vs. FD (**E**), and FSI vs. FW (**F**) for four fertilization treatments (control, NP, NPK, NPKMg) in an experimental apple orchard at Debrecen–Pallag, Hungary, over 2016–2022, on two apple cultivars ‘Pinova’ and ‘Golden Reinders’ (*n* = 14; 2 cultivars × 7 years). Explanations for control, NP, NPK and NKPMg are given in Table 2.

The linear regression analysis showed significant relationships for all six pair variables with *r* = 0.714–0.922 and *p* = 0.045–0.001 for the four fertilizer treatments. However, no differences were observed among the slope parameters for the six variable pairs among the control, NP, NPK, and NPKMg treatments as *t*-tests showed *p*-values ranging from 0.799 to 0.118. Slope parameters showed no significant differences between the two cultivars. FNT vs. TCSA relationships showed that most of the increasing values for the number of fruit per tree corresponded with increases in TCSA values (Figure 2A). In the case of TCSA vs. FW relationships, increasing TCSA values resulted in slight decreases in fruit weight for all four fertilizer treatments (Figure 2B). In the case of FNT vs. FY and CL vs. FY relationships, the majority of the number of fruit per tree values and crop load values were directly proportional to the increase in fruit yield for all four fertilizer treatments (Figure 2C,D). In the case of FD vs. FW relationships, increasing fruit diameter values resulted in sharp increases in fruit weight for all four fertilizer treatments (Figure 2E). In the case of FSI vs. FW relationships, the increase in the fruit scab incidence was not directly proportional to the increase in fruit weight (Figure 2F). Here, two clusters were separated. The cluster with values of zero or close to zero scab incidences includes the fruit of cv. ‘Pinova’.

## 4. Discussion

In this 7-year study, we evaluated the effects of four fertilizer treatments (control, NP, NPK, NPKMg) on vegetative, generative, and disease incidence parameters of two apple cultivars (‘Golden Reinders’ and ‘Pinova’). The effects of fertilizer treatments on the observed parameters were dependent on year and cultivars.

The fruit yield of the two cultivars was highly dependent on the differences in the yearly abiotic environmental factors, especially for cv. ‘Golden Reinders’. Severe fluctuations in yield from year to year are reported for several apple cultivars [47,48], and alternate bearing is a common feature of cv. ‘Golden Reinders’ in many growing areas [49,50]. Previous studies reported that alternate fruit bearing was not observed for cv. ‘Pinova’ [41], which was also confirmed by this 7-year study, as cv. ‘Pinova’ produced a more homogenous annual yield than cv. ‘Golden Reinders’ (Table 6, Table 7 and Table 8). However, our findings clearly demonstrate a biennial pattern of fruit production for cv. ‘Golden Reinders’, with reduced yields observed in 2017, 2019, and 2021, and improved yields in 2016, 2018, 2020, and 2022. This alternation coincides with varying levels of frost damage observed in our study. Our results align with previous research by Monselise and Goldschmidt [51], Atay et al. [51], and Netsawang et al. [48], which suggests that alternate bearing is influenced by multiple factors, including spring frost events for deciduous trees and drought stress during fruit set.

In previous studies on apples, fertilization has been reported to increase the TSCA values of the trees [52] or have no effect on TSCA values [53,54]. In our study, TCSA values increased annually in both cultivars (Table 5). However, the fertilization effect was predominantly observed in the latter half of the 7-year experimental period, particularly for cv. ‘Pinova’, which exhibits more balanced fruit-bearing characteristics. Pole et al. [52] demonstrated that nitrogen fertilization increased TSCA in apples, while our long-term results revealed a significant increase in TCSA with the combined NPKMg treatment in some years compared to NP or NPK fertilizer treatments. Conversely, previous studies have shown that fertilization did not affect the TCSA values of cultivars ‘Sampion’ and ‘Golden Delicious Reinders’ [53,54]. Differences in the effects of fertilization on TSCA are likely attributed to variations in plant density, tree age, ecological conditions, and fertilization dosages in our study. Our fertilization study revealed significant correlations between TSCA and the number of fruit per tree, and between TSCA and fruit weight, which were not explored in previous studies (Table 13 and Figure 2A,C).

The response of fruit yield to nutrient supply has often been inconsistent over the years or has yielded controversial results [52,53,54,55,56]. For instance, experiments involving nitrogen fertilization of apple orchards on fertile soil have frequently shown a lack of effectiveness of the fertilizer treatments [52,55,56], a trend that was also observed in this study in certain years when fertilization had no significant effect for either cultivar (Table 6). In our study, we observed optimum values of the nutrients P, K, and Mg (Table 1) in our sandy soil characterized by moderate fertility. This observation may elucidate the absence of detectable fertilization effects in certain years. Conversely, it has been noted that the optimal levels of P, K, and Mg in sandy soil are usually lower than those in ‘standard soils’ with higher fertility [39]. This finding potentially explains why the effects of fertilization became evident in other years of our study (Table 6). These latter results were consistent with findings by Stefanelli et al. [57], who reported positive effects of fertilization within moderate doses. In another trial, the response in yield was observed only after 10 years of fertilization when nitrogen, phosphorus, and potassium fertilizers were applied singly and in combination [58]. The authors demonstrated that while a single application of nitrogen reduced yield, combined NPK fertilizers significantly increased fruit yield [58]. These results align with our findings on TSCA values (Table 5) and fruit yield values in 2018 and 2022, where the highest yield response was observed in the NPK treatments for cv. ‘Golden Reinders’ (Table 6). Although our study did not involve the single application of nitrogen fertilizers, previous studies have indicated that the application of various doses of nitrogen had either a low or no effect on apple yield [59,60], but it improved shoot extension growth in cv. ‘Golden Reinders’ apple trees [59]. In addition, to our knowledge, previous studies have not demonstrated correlations among fruit yield parameters under fertilization treatments. Overall, our results on fruit yield indicate that the effects of combined NP, K, and Mg nutrients on fruit yield vary depending on several factors, including seasonal features, cultivar characteristics, and nutrient type combinations. This suggests that a reliable yield response to fertilization can be determined after long-term (min. 10 years) experimentation and under specific orchard ecological conditions.

In many cases, external features such as fruit size and fruit weight hold greater importance to consumers than the internal traits [61]. The cultivar ‘Pinova’ typically exhibits an average fruit weight of 175 g and a fruit diameter of 75 mm [41,62]. In our study, under various fertilization treatments, cv. ‘Pinova’ produced fruits of 133–203 g, with diameters ranging from 69.1 to 77.9 mm. These values align with findings from a study utilizing nanotechnology-based foliar fertilizers [63]. For cv. ‘Golden Reinders’, the fruit diameter consistently reached 70 mm each year with fruit weights ranging from 145 to 238 g, consistent with results reported in other studies [20,64]. Bielicki and Pasko [65] reported a yield of 42.2 kg tree^−1^ for cv. ‘Golden Reinders’ on M.26 rootstock. Notably, our fertilization trial yielded higher values for cv. ‘Golden Reinders’, such as in 2022, when fruit yield reached 64.0–69.1 kg tree^−1^ (Table 6). Our study indicates that fertilization treatments were effective in improving fruit weight and size in both cultivars, as evidenced by generally smaller fruit diameters and weights observed on control trees in most years. This was also revealed with a positive correlation between fruit weight and fruit diameter (Table 13, Figure 2E). Our findings are consistent with the results of Zhao et al. [66] and Zijian et al. [67], who demonstrated that fertilization increased fruit quality, including mean fruit weight and diameter. Although we employed identical fertilization regimes for both cultivars in our study, owing to their comparable growth characteristics, it is conceivable that cultivar-specific fertilization strategies could offer greater benefits to growers, as evidenced by prior research [68,69].

Disease-tolerant/resistant and -susceptible cultivars exhibited differing levels of susceptibility in terms of FSI and PMIS in this study (Table 11 and Table 12), consistent with findings from previous studies [70,71,72]. Our fertilizer treatments clearly demonstrated that complete nutrient supply (NPKMg) consistently reduced disease incidence for both diseases across most cases and years, especially on the disease-susceptible cv. ‘Golden Reinders’ (Table 11 and Table 12). This observation is consistent with prior research, indicating that optimal uptake of NPK and Mg nutrients by apple trees enhances tolerance to diseases, while nutrient imbalances may increase susceptibility to diseases and pests [26,29,30]. In this study, we adhered to a standard fungicide spray regimen in accordance with integrated fruit production guidelines. It is plausible that the disparities would have been more pronounced in the absence or reduction of efficient fungicidal sprays. We also need to note that some nutrients, such as N, can increase apple tree canker development caused by *N. ditissima* [37,38]. In addition, N excess induces vigorous shoot growth in apple trees, which elevates the susceptibility of shoots to *P. leucotricha* [35,38] and reduces leaf resistance to *V. inaequalis* [31,32]. However, for instance, N fertilization in autumn can enhance the decomposition of leaf litter, thereby reducing the overwintering inoculum of *V. inaequalis* and consequently decreasing scab incidence in the following spring [35,73,74,75,76,77]. Our study also revealed that the effect of fertilization on disease incidence was notably lower in the disease-tolerant cultivar ‘Pinova’ (Table 11 and Table 12). This can be attributed to the inherently lower disease incidences of the disease-resistant cultivar in the control treatment, a phenomenon also observed in previous studies investigating cultivar susceptibilities to diseases [40,41,70,78]. However, while fertilization treatments for FSI and PMIS showed little significant correlation with other measured parameters and between each other (Table 13), we found that only fruit weight exhibited a significant positive correlation with fruit scab incidence (Figure 2F). This finding is consistent with practical experience, as scabbed fruits tend to be smaller in size and weight compared to healthy fruits [78,79].

## 5. Conclusions

Our study demonstrated that the multiyear application of fertilizer combinations could effectively increase TCSA and yield parameters in two apple cultivars while also reducing fungal disease incidences in the disease-susceptible apple cultivar grown in sandy soil with moderate fertility, under Central-European continental climate conditions. However, the effect could be largely influenced by environmental and meteorological conditions of the year, as well as by the alternate bearing features of the cultivar. Furthermore, the outcome of unbalanced nutrient addition often did not differ significantly from that of balanced fertilizer combinations. Our study suggests a recommendation for orchard-specific practices, which necessitates thorough local investigations for several years. This duration allows for a comprehensive understanding of each tree’s specific requirements within the given environmental circumstances.

## Figures and Tables

**Table 2:** Four fertilizer treatments applied and doses of fertilizer active ingredients (nitrogen—N; phosphorus—P_2_O_5_; potassium—K_2_O; and magnesium—MgO) in the experimental apple orchard at Debrecen–Pallag, Hungary, from 2016 to 2022.

	N (kg ha^−1^)	P_2_O_5_ (kg ha^−1^)	K_2_O (kg ha^−1^)	MgO (kg ha^−1^)
**Control**	0	0	0	0
**NP**	60	80	0	0
**NPK**	60	80	100	0
**NPKMg**	60	80	100	30

**Table 3:** Application time (dd mm yyyy) of the fertilizers (nitrogen—N; phosphorus—P; potassium—K; and magnesium—Mg) in the experimental apple orchard at Debrecen–Pallag, Hungary, from 2016 to 2022.

Date of Fertilizing	Supplied Elements
7 April 2016	N, P, K, Mg
16 November 2016	P, K, Mg
22 March 2017	N
15 November 2017	P, K, Mg
29 March 2018	N
14 November 2018	P, K, Mg
20 March 2019	N
15 November 2019	P, K, Mg
17 March 2020	N
18 November 2020	P, K, Mg
19 March 2021	N
17 November 2021	P, K, Mg
13 March 2022	N

**Table 4:** Analysis of variance for the effects of cultivars (‘Pinova’ and ‘Golden Reinders’), fertilizer treatments (Control, NP, NPK, NPKMg) and years (2016–2022) on trunk cross-sectional area (TCSA), fruit yield (FY), number of fruit per tree (FNT), crop load (CL), fruit diameter (FD), fruit weight (FW), fruit scab incidence (FSI), and powdery mildew incidence on shoot (PMIS). Significant values (*p* < 0.05) are indicated with bold figures.

Source of Variation		TCSA			FY			FNT			CL	
	df	MS	*p*	df	MS	*p*	df	MS	*p*	df	MS	*p*
Cultivar (C)	1	208	0.0059	1	58.2	**0.049**	1	1242	**0.0380**	1	0.052	**0.049**
Fertilizer (F)	3	319	**<0.001**	3	142.5	**0.037**	3	5759	**0.033**	3	0.062	**0.021**
Year (Y)	6	2556	**<0.001**	5	1861	**<0.001**	5	137,180	**<0.001**	5	0.438	**<0.001**
C × F	3	428	0.069	3	30.8	0.518	3	2249	0.261	3	0.017	0.335
C × Y	6	3.55	0.949	5	500	0.058	5	30,004	0.054	5	0.049	0.052
F × Y	18	22.1	0.154	15	28.3	0.729	15	2001	0.301	15	0.193	0.671
C × F × Y	18	13.5	0.395	15	39.1	0.532	15	1519	0.435	15	0.158	0.487
Total	55			47			47			47		
		**FD**			**FW**			**FSI**			**PMIS**	
	**df**	**MS**	***p***	**df**	**MS**	***p***	**df**	**MS**	***p***	**df**	**MS**	***p***
Cultivar (C)	1	6.04	**0.033**	1	386	**0.042**	1	851	**<0.001**	1	70.3	**<0.001**
Fertilizer (F)	3	4.28	**0.031**	3	198	**0.217**	3	3.38	**<0.001**	3	3.49	**<0.001**
Year (Y)	6	40.2	**<0.001**	5	1598	**<0.001**	4	13.5	**<0.001**	5	5.22	**<0.001**
C × F	3	1.27	0.356	3	31.5	0.850	3	3.67	0.059	3	0.064	0.421
C × Y	6	17.8	0.051	5	922	0.054	4	2.78	0.051	5	0.175	0.057
F × Y	18	2.38	0.071	15	193	0.181	12	0.218	0.118	15	0.074	0.376
C × F × Y	18	1.09	0.523	15	119	0.439	12	0.106	0.241	15	0.063	0.412
Total	55			47			39			47		

df: degrees of freedom. *p*: the probability values associated with the F-tests. MS: mean squares.

**Table 5:** Trunk cross-sectional area (TCSA, cm^2^) of two apple cultivars (‘Pinova’ and ‘Golden Reinders’) in four fertilizer treatments (Control, NP, NPK, NPKMg) (Debrecen–Pallag, Hungary, 2016–2022). In brackets, the difference in each year was presented with 2016 as the reference point (0), and then Y2-Y1, Y3-Y2, etc. ns: nonsignificant.

Treatments	2016	2017	2018	2019	2020	2021	2022	Overall (Year)
**Golden Reinders**								
**Control**	34.4 (0)	50.9 (16.5)	60.7 (9.8)	69.8 ab ^b^ (9.1)	74.0 ab (4.3)	83.6 (9.5)	91.9 ab (8.3)	66.5
**NP**	35.6 (0)	38.8 (3.2)	45.8 (7.0)	53.0 a (7.2)	59.5 a (6.5)	63.1 (3.6)	72.9 a (9.8)	52.7
**NPK**	31.4 (0)	47.9 (16.5)	53.5 (5.4)	65.7 ab (12.4)	70.1 ab (4.4)	78.7 (8.6)	86.1 ab (7.3)	61.9
**NPKMg**	33.8 (0)	48.3 (14.5)	60.9 (12.6)	75.2 b (1.7)	78.1 b (15.5)	87.4 (9.4)	92.9 b (5.5)	68.1
**LSD_0.05_** ^a^	ns	ns	ns	20.4	18.2	ns	19.9	ns
**Pinova**								
**Control**	38.3 ab (0)	54.9 ab (16.6)	63.0 ab (8.1)	71.2 (8.2)	75.7 ab (4.5)	81.9 b (6.2)	87.2 ab (5.3)	67.5 ab
**NP**	48.0 b (0)	61.4 b (13.4)	67.1 b (5.7)	79.0 (11.9)	82.5 b (3.5)	89.2 b (6.7)	95.3 b (6.1)	74.7 b
**NPK**	32.0 a (0)	44.0 a (12.0)	51.1 a (7.1)	57.4 (6.3)	62.1 a (4.7)	65.9 a (3.8)	72.0 a (6.1)	54.9 a
**NPKMg**	37.3 ab (0)	52.9 ab (15.6)	59.4 ab (6.5)	68.5 (9.1)	84.0 b (15.6)	91.6 b (7.6)	102.5 b (10.9)	70.9 b
**LSD_0.05_**	12.0	13.2	14.8	ns	15.4	16.7	17.3	14.9
**Overall (cultivars)**								
**Control**	36.3 (0)	52.9 (16.5)	61.8 (9.0)	70.5 (8.6)	74.9 ab (4.4)	82.7 ab (7.9)	89.6 ab (6.8)	67.0
**NP**	41.8 (0)	50.1 (8.3)	56.5 (6.4)	66.0 (9.5)	71.0 ab (5.0)	76.1 ab (5.1)	84.1 ab (8.0)	63.7
**NPK**	31.7 (0)	45.9 (14.2)	52.3 (6.3)	61.5 (9.3)	66.1 a (4.6)	72.3 a (6.2)	79.1 a (6.7)	58.4
**NPKMg**	35.6 (0)	50.6 (15.0)	60.1 (9.6)	71.8 (5.4)	81.0 b (15.5)	89.5 b (8.5)	97.7 b (8.2)	69.5
**LSD_0.05_**	ns	ns	ns	ns	14.8	16.8	18.2	ns
**Overall (treatments)**								
**Golden R.**	33.8 (0)	46.5 (12.7)	55.2 (8.7)	65.9 (7.6)	70.4 (7.7)	78.2 (7.8)	85.9 (7.8)	62.3
**Pinova**	38.9 (0)	53.3 (14.4)	60.2 (6.9)	69.0 (8.9)	76.1 (7.1)	82.2 (6.1)	89.3 (7.1)	67.0
**LSD_0.05_**	ns	ns	ns	ns	ns	ns	ns	ns

^a^ Differences among treatments are represented by LSD_0.05_ values at *p* = 0.05. ^b^ Values coupled with different letters are significantly different at *p* = 0.05 according to LSD *t*-tests. If there are no letters beside the values, it indicates that there are no significant differences between the treatment values.

**Table 6:** Total fruit yield (kg tree^−1^) of two apple cultivars (‘Pinova’ and ‘Golden Reinders’) in four fertilizer treatments (control, NP, NPK, NPKMg) (Debrecen–Pallag, Hungary, 2016–2022). ns: nonsignificant.

Treatments	2016	2017	2018	2019	2020	2021	2022	Overall (Year)
**Golden Reinders**								
**Control**	38.7	15.5 ab ^b^	44.0 ab	4.6	52.7	- ^c^	45.7 a	33.5
**NP**	36.9	21.4 b	35.3 a	9.2	54.0	-	64.0 b	36.8
**NPK**	44.0	10.1 a	60.1 b	2.8	64.8	-	69.1 b	41.8
**NPKMg**	32.5	15.1 ab	49.8 ab	8.0	62.9	-	66.7 b	39.2
**LSD_0.05_** ^a^	ns	11.2	24.1	ns	ns	-	17.6	ns
**Pinova**								
**Control**	28.4 a	25.8	26.7 a	30.6 a	46.1	42.5	44.8 a	35.0
**NP**	39.7 b	32.0	42.5 b	33.1 ab	48.0	42.8	63.2 b	43.1
**NPK**	39.6 b	34.8	38.5 ab	39.9 b	43.4	46.0	51.4 ab	41.9
**NPKMg**	38.3 b	23.7	44.0 b	31.3 ab	45.7	44.5	53.0 ab	40.1
**LSD_0.05_**	9.7	ns	14.5	9.2	ns	ns	18.1	ns
**Overall (cultivars)**								
**Control**	33.5	20.7	35.3 a	17.6	49.4	42.5	45.2 a	34.9
**NP**	38.3	26.7	38.9 ab	21.2	51.0	42.8	63.6 b	40.3
**NPK**	41.8	22.4	49.3 b	21.4	54.1	46.0	60.2 ab	42.2
**NPKMg**	35.4	19.4	46.9 ab	19.6	54.3	44.5	59.8 ab	40.0
**LSD_0.05_**	ns	ns	13.9	ns	ns	ns	17.9	ns
**Overall (treatments)**								
**Golden R.**	38.0	15.5 a	47.3	6.1 a	58.6	-	61.3	37.8
**Pinova**	36.5	29.1 b	37.9	33.7 b	45.8	43.9	53.1	40.0
**LSD_0.05_**	ns	13.2	ns	6.0	ns	-	ns	ns

^a^ Differences among treatments are represented by LSD_0.05_ values at *p* = 0.05. ^b^ Values coupled with different letters are significantly different at *p* = 0.05 according to LSD *t*-tests. If there are no letters beside the values, it indicates that there are no significant differences between the treatment values. ^c^ ‘-’ No data available for cv. ‘Golden Reinders’ in 2021 due to frost damage.

**Table 7:** Number of fruit per tree of two apple cultivars (‘Pinova’ and ‘Golden Reinders’) in four fertilizer treatments (control, NP, NPK, NPKMg) (Debrecen–Pallag, Hungary, 2016–2022). ns: nonsignificant.

Treatments	2016	2017	2018	2019	2020	2021	2022	Overall (Year)
**Golden Reinders**								
**Control**	193 ab ^b^	71 ab	232 a	25 ab	491 ab	- ^c^	381 a	232
**NP**	192 ab	129 a	192 a	45 b	406 a	-	393 ab	226
**NPK**	219 b	48 b	369 b	14 a	612 b	-	445 ab	284
**NPKMg**	154 a	71 ab	264 a	37 b	548 ab	-	478 b	259
**LSD_0.05_** ^a^	58	80	104	21	198	-	84	ns
**Pinova**								
**Control**	143 a	136	174 a	174 a	333	321	273 a	222
**NP**	219 b	181	267 b	194 ab	300	362	379 b	272
**NPK**	222 b	173	228 ab	245 b	387	353	304 ab	273
**NPKMg**	203 b	129	254 ab	177 ab	345	380	324 ab	259
**LSD_0.05_**	50	ns	87	57	ns	ns	74	ns
**Overall (cultivars)**								
**Control**	168 a	104	203 a	99	412	321	327 a	233
**NP**	205 ab	155	230 ab	120	353	362	386 ab	259
**NPK**	220 b	110	298 b	129	500	353	374 ab	283
**NPKMg**	179 ab	100	259 ab	107	447	380	401 b	267
**LSD_0.05_**	51	ns	94	ns	ns	ns	73	ns
**Overall (treatments)**								
**Golden R.**	189	80 a	264	30 a	514 b	-	424 b	250
**Pinova**	197	155 b	231	198 b	341 a	353	320 a	256
**LSD_0.05_**	ns	73	ns	29	161	-	96	ns

^a^ Differences among treatments are represented by LSD_0.05_ values at *p* = 0.05. ^b^ Values coupled with different letters are significantly different at *p* = 0.05 according to LSD *t*-tests. If there are no letters beside the values, it indicates that there are no significant differences between the treatment values. ^c^ ‘-’ No data available for cv. ‘Golden Reinders’ in 2021 due to frost damage.

**Table 8:** Crop load (kg cm^−2^) of two apple cultivars (‘Pinova’ and ‘Golden Reinders’) in four fertilizer treatments (control, NP, NPK, NPKMg) (Debrecen–Pallag, Hungary, 2016–2022). ns: nonsignificant.

Treatments	2016	2017	2018	2019	2020	2021	2022	Overall (Year)
**Golden Reinders**								
**Control**	0.96 ab ^b^	0.34	0.78 a	0.09 ab	1.03	- ^c^	0.83	0.67
**NP**	1.21 b	0.60	0.78 a	0.16 b	0.88	-	0.91	0.75
**NPK**	1.25 b	0.21	1.15 b	0.05 a	1.05	-	0.87	0.76
**NPKMg**	0.83 a	0.32	0.91 ab	0.11 ab	0.86	-	0.96	0.66
**LSD_0.05_** ^a^	ns	ns	0.48	0.09	ns	-	ns	ns
**Pinova**								
**Control**	0.67 a	0.47	0.42 a	0.47 a	0.64	0.52	0.57 a	0.54 a
**NP**	0.74 ab	0.58	0.70 ab	0.51 ab	0.60	0.49	0.63 a	0.61 ab
**NPK**	1.00 b	0.78	0.76 b	0.70 b	0.73	0.71	0.74 b	0.77 b
**NPKMg**	0.90 ab	0.44	0.73 b	0.49 ab	0.65	0.58	0.59 a	0.63 ab
**LSD_0.05_**	0.32	ns	0.29	0.22	ns	ns	ns	0.22
**Overall (cultivars)**								
**Control**	0.81	0.41	0.60 a	0.28	0.83	0.52	0.70	0.59
**NP**	0.97	0.59	0.74 ab	0.34	0.74	0.49	0.77	0.66
**NPK**	1.13	0.50	0.95 b	0.37	0.89	0.71	0.80	0.77
**NPKMg**	0.86	0.38	0.82 ab	0.30	0.75	0.58	0.77	0.64
**LSD_0.05_**	ns	ns	0.34	ns	ns	ns	ns	ns
**Overall (treatments)**								
**Golden R.**	1.06	0.37	0.91	0.10 a	0.95 b	-	0.89	0.89
**Pinova**	0.83	0.57	0.65	0.54 b	0.65 a	0.58	0.63	0.64
**LSD_0.05_**	ns	ns	ns	0.24	0.29	-	ns	ns

^a^ Differences among treatments are represented by LSD_0.05_ values at *p* = 0.05. ^b^ Values coupled with different letters are significantly different at *p* = 0.05 according to LSD *t*-tests. If there are no letters beside the values, it indicates that there are no significant differences between the treatment values. ^c^ ‘-’ No data available for cv. ‘Golden Reinders’ in 2021 due to frost damage.

**Table 9:** Fruit diameter (mm) of two apple cultivars (‘Pinova’ and ‘Golden Reinders’) in four fertilizer treatments (control, NP, NPK, NPKMg) (Debrecen–Pallag, Hungary, 2016–2022). ns: nonsignificant.

Treatments	2016	2017	2018	2019	2020	2021	2022	Overall (Year)
**Golden Reinders**								
**Control**	79.3 ab ^b^	78.5 ab	75.6 a	74.4 a	70.5	- ^c^	73.6	75.3
**NP**	79.0 a	75.9 a	78.2 b	72.5 a	71.1	-	74.1	75.1
**NPK**	80.1 ab	80.7 b	76.1 ab	74.7 a	70.1	-	72.4	75.7
**NPKMg**	83.0 b	78.6 ab	78.6 b	77.9 b	70.9	-	72.9	77.0
**LSD_0.05_** ^a^	2.8	3.5	2.5	3.1	ns	-	ns	ns
**Pinova**								
**Control**	76.6	76.4	71.5 a	73.0 a	73.3	69.1 a	76.7 b	73.8
**NP**	77.1	75.7	73.3 ab	75.4 b	74.8	71.3 b	75.5 ab	74.7
**NPK**	77.0	76.1	72.8 ab	74.3 ab	74.9	69.8 ab	73.6 a	74.1
**NPKMg**	77.9	76.3	75.7 b	75.2 b	74.8	70.0 ab	73.8 a	74.8
**LSD_0.05_**	ns	ns	2.9	2.1	ns	2.1	2.7	ns
**Overall (cultivars)**								
**Control**	77.9 a	77.5	73.5 a	73.7 a	71.9	69.1 a	75.2	74.1
**NP**	78.1 ab	75.8	75.8 ab	73.9 ab	73.0	71.3 b	74.8	74.7
**NPK**	78.6 ab	78.4	74.5 ab	74.5 ab	72.5	69.8 ab	73.0	74.5
**NPKMg**	80.5 b	77.5	77.1 b	76.6 b	72.9	70.0 ab	73.4	75.4
**LSD_0.05_**	2.5	ns	2.9	2.8	ns	2.1	ns	ns
**Overall (treatments)**								
**Golden R.**	80.4	78.4	77.1	74.9	70.7	-	73.3	75.8
**Pinova**	77.1	76.1	73.3	74.5	74.5	70.0	74.9	74.4
**LSD_0.05_**	3.2	ns	3.5	ns	2.1	-	ns	ns

^a^ Differences among treatments are represented by LSD_0.05_ values at *p* = 0.05. ^b^ Values coupled with different letters are significantly different at *p* = 0.05 according to LSD *t*-tests. If there are no letters beside the values, it indicates that there are no significant differences between the treatment values. ^c^ ‘-’ No data available for cv. ‘Golden Reinders’ in 2021 due to frost damage.

**Table 10:** Fruit weight (g) of two apple cultivars (‘Pinova’ and ‘Golden Reinders’) in four fertilizer treatments (control, NP, NPK, NPKMg) (Debrecen–Pallag, Hungary, 2016–2022). ns: nonsignificant.

Treatments	2016	2017	2018	2019	2020	2021	2022	Overall (Year)
**Golden Reinders**								
**Control**	196 a	215 b	190 a	187 b	154	- ^c^	176	186
**NP**	199 a	190 a	208 b	152 a	153	-	181	180
**NPK**	203 a	212 b	189 a	201 b	145	-	169	187
**NPKMg**	238 b	222 b	206 b	155 a	150	-	178	191
**LSD_0.05_** ^a^	21	16	11	21	ns	-	ns	ns
**Pinova**								
**Control**	179 a	185 ab	159 a	169 a	170 a	133 a	203 b	171
**NP**	184 ab ^b^	182 a	168 a	180 ab	172 ab	154 b	190 ab	175
**NPK**	191 ab	199 ab	164 a	182 b	178 ab	147 b	177 a	177
**NPKMg**	198 b	202 b	181 b	182 b	183 b	145 b	176 a	181
**LSD_0.05_**	17	18	12	12	12	11	21	ns
**Overall (cultivars)**								
**Control**	188 a	200 ab	175 a	178 ab	162	133 a	190	175
**NP**	191 a	186 a	188 bc	166 a	163	154 b	185	176
**NPK**	197 ab	205 b	177 ab	191 b	162	147 b	173	179
**NPKMg**	218 b	212 b	193 c	168 a	166	145 b	177	183
**LSD_0.05_**	21	18	12	20	ns	11	ns	ns
**Overall (treatments)**								
**Golden R.**	209 b	210 b	198 b	173	151 a	-	176	186
**Pinova**	188 a	192 a	168 a	178	176 b	145	186	176
**LSD_0.05_**	20	17	15	ns	16	-	ns	ns

^a^ Differences among treatments are represented by LSD_0.05_ values at *p* = 0.05. ^b^ Values coupled with different letters are significantly different at *p* = 0.05 according to LSD *t*-tests. If there are no letters beside the values, it indicates that there are no significant differences between the treatment values. ^c^ ‘-’ No data available for cv. ‘Golden Reinders’ in 2021 due to frost damage.

**Table 11:** Apple scab incidence (%) on fruit of two apple cultivars (the disease-susceptible cv. ‘Golden Reinders’ and the apple scab and powdery mildew-tolerant cv. ‘Pinova’) in four fertilizer treatments (control, NP, NPK, NPKMg) (Debrecen–Pallag, Hungary, 2016–2022). ns: nonsignificant.

Treatments	2016	2017	2018	2019	2020	2021	2022	Overall (Year)
**Golden Reinders**								
**Control**	13.5 b ^b^	12.5 b	8.7	8.6	- ^c^	-	10.5 b	10.8
**NP**	14.0 b	12.3 b	9.1	8.3	-	-	10.1 ab	10.8
**NPK**	11.7 ab	10.2 ab	8.4	8.3	-	-	9.6 ab	9.6
**NPKMg**	10.6 a	9.5 a	7.5	7.1	-	-	8.3 a	8.6
**LSD_0.05_^a^**	2.8	2.6	ns	ns	-	-	2.1	ns
**Pinova**								
**Control**	2.3 b	1.5	0	0	-	0	1	0.8
**NP**	1.8 ab	1.1	0	0	-	0	0.7	0.6
**NPK**	1.5 a	1	0	0	-	0	0.5	0.5
**NPKMg**	1.5 a	1	0	0	-	0	0.5	0.5
**LSD_0.05_**	0.7	ns	ns	ns	-	ns	ns	ns
**Overall (cultivars)**								
**Control**	7.9 b	7.0 b	4.4	4.3	-	0.0	5.8	4.9
**NP**	7.9 b	6.7 ab	4.6	4.2	-	0.0	5.4	4.8
**NPK**	6.6 ab	5.6 ab	4.2	4.2	-	0.0	5.1	4.3
**NPKMg**	6.1 a	5.3 a	3.8	3.6	-	0.0	4.4	3.8
**LSD_0.05_**	1.7	1.6	ns	ns	-	ns	ns	ns
**Overall (treatments)**								
**Golden R.**	12.5 b	11.1 b	8.4 b	8.1 b	-	-	9.6 b	9.9 b
**Pinova**	1.8 a	1.2 a	0.0 a	0.0 a	-	0.0	0.7 a	0.6 a
**LSD_0.05_**	1.5	1.1	1.3	1.5	-	-	0.7	1.2

^a^ Differences among treatments are represented by LSD_0.05_ values at *p* = 0.05. ^b^ Values coupled with different letters are significantly different at *p* = 0.05 according to LSD *t*-tests. If there are no letters beside the values, it indicates that there are no significant differences between the treatment values. ^c^ ‘-’ missing data in 2020, and no data available for cv. ‘Golden Reinders’ in 2021 due to frost damage.

**Table 12:** Apple powdery mildew incidence (%) on shoots of two apple cultivars (‘Pinova’ and ‘Golden Reinders’) in four fertilizer treatments (control, NP, NPK, NPKMg) (Debrecen–Pallag, Hungary, 2016–2022). ns: nonsignificant.

Treatments	2016	2017	2018	2019	2020	2021	2022	Overall (Year)
**Golden Reinders**								
**Control**	4.7	5.8 b ^b^	7.3 b	5.6	- ^c^	5.0 b	6.8 b	5.9 b
**NP**	4.9	5.6 ab	6.9 ab	5.5	-	4.7 ab	6.3 ab	5.7 ab
**NPK**	4.3	5.1 ab	6.1 ab	5.1	-	3.8 ab	5.5 ab	5.0 ab
**NPKMg**	4.1	4.1 a	5.6 a	4.9	-	3.4 a	5.1 a	4.5 a
**LSD_0.05_^a^**	ns	1.5	1.3	ns	-	1.4	1.4	1.4
**Pinova**								
**Control**	2.3	3.5	4.6 b	3.6	-	2.3	3.5	3.3
**NP**	2.0	3.1	4.4 ab	3.3	-	2.1	4.0	3.2
**NPK**	1.5	2.7	3.2 ab	3.2	-	1.8	3.2	2.6
**NPKMg**	1.3	2.6	2.7 a	2.8	-	1.6	2.8	2.3
**LSD_0.05_**	ns	ns	1.5	ns	-	ns	ns	ns
**Overall (cultivars)**								
**Control**	3.5	4.7	6.0 b	4.6	-	3.7	5.2	4.6
**NP**	3.5	4.4	5.7 ab	4.4	-	3.4	5.2	4.4
**NPK**	2.9	3.9	4.7 ab	4.2	-	2.8	4.4	3.8
**NPKMg**	2.7	3.4	4.2 a	3.9	-	2.5	4.0	3.4
**LSD_0.05_**	ns	ns	1.5	ns	-	ns	ns	ns
**Overall (treatments)**								
**Golden R.**	4.5 b	5.2 b	6.5 b	5.3 b	-	4.2 b	5.9 b	5.3 b
**Pinova**	1.8 a	3.0 a	3.7 a	3.2 a	-	2.0 a	3.4 a	2.8 a
**LSD_0.05_**	1.6	1.7	1.5	1.6	-	1.6	1.7	1.6

^a^ Differences among treatments are represented by LSD_0.05_ values at *p* = 0.05. ^b^ Values coupled with different letters are significantly different at *p* = 0.05 according to LSD *t*-tests. If there are no letters beside the values, it indicates that there are no significant differences between the treatment values. ^c^ ‘-‘ missing data in 2020.

**Table 13:** Pearson’s correlation coefficients (*r*) amongst eight measures for four fertilizer treatments (control, NP, NKP, NKPMg) in an experimental apple orchard at Debrecen–Pallag, Hungary, over 2016–2022, on two apple cultivars ‘Pinova’ and ‘Golden Reinders’. Measured eight parameters: trunk cross-sectional area (TCSA), fruit yield (FY), number of fruit per tree (FNT), crop load (CL), fruit diameter (FD), fruit weight (FW), fruit scab incidence (FSI), and powdery mildew incidence on shoots (PMIS). Bold figures represent significant (*p* < 0.05) correlation coefficient values.

**Overall**	**TCSA**	**FY**	**FNT**	**CL**	**FD**	**FW**	**FSI**
**FY**	0.38						
**FNT**	**0.55**	**0.95**					
**CL**	−0.26	**0.74**	0.41				
**FD**	−0.39	−0.17	−0.39	0.09			
**FW**	**−0.52**	−0.18	−0.37	0.16	**0.79**		
**FSI**	−0.33	−0.16	−0.21	0.16	0.36	**0.52**	
**PMIS**	0.11	−0.05	−0.08	0.01	0.41	0.28	−0.46
**Pinova**	**TCSA**	**FY**	**FNT**	**CL**	**FD**	**FW**	**FSI**
**FY**	**0.56**						
**FNT**	**0.67**	**0.88**					
**CL**	−0.44	**0.49**	0.09				
**FD**	−0.41	−0.24	**−0.65**	0.19			
**FW**	**−0.49**	−0.15	**−0.51**	0.26	**0.81**		
**FSI**	−0.45	−0.22	−0.44	0.35	0.42	**0.56**	
**PMIS**	0.28	−0.05	−0.16	−0.25	0.43	0.08	−0.32
**Golden R.**	**TCSA**	**FY**	**FNT**	**CL**	**FD**	**FW**	**FSI**
**FY**	0.29						
**FNT**	**0.49**	**0.97**					
**CL**	−0.15	**0.85**	**0.76**				
**FD**	**−0.54**	−0.23	−0.38	−0.14			
**FW**	**−0.56**	−0,13	−0.23	0.13	**0.76**		
**FSI**	−0.46	0.03	−0.02	0.38	0.39	**0.49**	
**PMIS**	0.39	0.17	0.23	0.18	−0.44	0.31	−0.22

## Data Availability

Data will be provided for other scientists upon reasonable request.

## Supplementary Materials

The following supporting information can be downloaded at: https://www.mdpi.com/article/10.3390/plants13091217/s1, Supplementary Table S1: The mean and minimum temperatures and precipitation (Debrecen-Pallag, Hungary, 2016–2022).
