# Supplementary material for: Impacts of N-P-K-Mg Fertilizer Combinations on Tree Parameters and Fungal Disease Incidences in Apple Cultivars with Varying Disease Susceptibility
Source: Plants (Basel). 2024 Apr 28;13(9):1217. doi: 10.3390/plants13091217 (PMC11085188; doi:10.3390/plants13091217)
Supplement: Supplementary file 1 [file plants-13-01217-s001.zip › plants-2933333-supplementary.pdf]

Supplementary material:

Table S1. The mean and minimum temperatures and precipitation (Debrecen-Pallag, Hungary, 2016-2022).

Months	Mean temperature (°C)							Minimum temperature (°C)							Precipitation (mm)						
	2016	2017	2018	2019	2020	2021	2022	2016	2017	2018	2019	2020	2021	2022	2016	2017	2018	2019	2020	2021	2022
January	-1.7	-6.0	2.6	-1.3	-1.3	1.4	-0.2	-15.7	-17.9	-8.0	-13.4	-9.0	-11.7	-11.4	63	21	27	24	24	37	8
February	6.1	2.1	0.4	3.4	4.6	2.3	4.1	-4.1	-5.0	-13.4	-6.1	-7.9	-12.8	-5.3	91	40	58	7	53	39	13
March	7.0	9.2	3.6	8.7	7.0	5.3	5.2	-6.2	-2.6	-17.0	-5.6	-4.1	-7.9	-9.0	48	28	69	8	39	21	10
April	13.3	10.7	16.5	13.3	11.5	9.0	9.7	-1.0	-0.7	4.2	1.2	-7.5	-4.6	-2.6	15	51	25	47	17	45	49
May	16.5	17.2	20.2	14.7	14.8	15.1	17.9	3.0	3.5	10.5	5.2	0.8	1.6	3.7	44	27	49	69	33	63	39
June	21.1	22.2	21.3	23.7	20.5	22.8	23.1	7.1	9.6	7.1	11.4	6.6	7.8	8.9	118	64	65	31	147	8	13
July	22.3	22.3	22.9	21.9	21.8	25.0	23.9	9.6	9.1	7.9	8.3	9.7	14.1	7.5	78	69	36	56	101	52	38
August	20.8	23.2	24.1	23.5	23.2	21.2	23.8	7.9	7.7	11.0	9.2	10.2	8.3	13.7	49	61	25	18	29	31	10
September	17.6	16.4	17.6	17.0	17.9	16.4	15.8	3.4	2.0	2.0	1.1	6.5	3.1	3.6	62	78	17	22	52	18	153
October	9.7	10.8	12.7	12.3	12.3	9.5	12.2	-0.8	-3.4	-1.3	-2.4	2.6	-3.4	-1.8	89	38	13	16	73	13	8
November	4.6	5.7	6.9	9.8	5.2	5.4	6.8	-5.1	-5.1	-10.8	-3.6	-5.3	-6.1	-1.6	56	57	39	81	15	30	49
December	-1.8	2.9	0.7	6.4	4.5	1.3	2.6	-9.5	-2.6	-12.1	-3.2	-6.4	-8.7	-7.3	6	102	44	49	28	39	92
Sum	-	-	-	-	-	-	-	-	-	-	-	-	-	-	720	636	466	429	611	397	482
